# Anisotropic Compressive Behavior of Functionally Density Graded Aluminum Foam Prepared by Controlled Melt Foaming Process

**DOI:** 10.3390/ma11122470

**Published:** 2018-12-05

**Authors:** Bingbing Zhang, Shuangqi Hu, Zhiqiang Fan

**Affiliations:** 1 School of Environment and Safety Engineering, North University of China, Taiyuan 030051, China; nomienn@126.com; 2 School of Science, North University of China, Taiyuan 030051, China; fanzhq@nuc.edu.cn

**Keywords:** density graded aluminum foams, anisotropy, mechanical behavior, deformation mechanism, digital image correlation

## Abstract

Aluminum foams with a functionally graded density have exhibited better impact resistance and a better energy absorbing performance than aluminum foams with a uniform density. Nevertheless, the anisotropic compression behavior caused by the graded density has scarcely been studied. In this paper, a density graded aluminum foam (FG) was prepared by a controlled foaming process. The effect of density anisotropy on the mechanical behavior of FGs was investigated under quasi-static compression and a low-velocity impact. Digital image correlation (DIC) and numerical simulation techniques were used to identify deformation mechanisms at both macro and cell levels. Results show that transverse compression on FGs lead to a higher collapse strength but also to a lower energy absorption, due to the significant decrease in densification strain and plateau stress. The deformation behavior of FGs under longitudinal compression was dominated by the progressive extension of the deformation bands. For FGs under transverse compression, the failure mode of specimens was characterized by multiple randomly distributed deformation bands. Moreover, the transverse compression caused more deformation on cells, through tearing and lateral stretching, because of the high lateral strain level in the specimens. It was concluded that the transverse compression of FGs lead to a lower plateau stress and a lower cell usage, thus resulting in a poorer energy absorption efficient; this constitutes a key factor which should be taken into consideration in structural design.

## 1. Introduction

Aluminum foams have found many potential applications in efficient constructions, lightweight structures, heat insulation and acoustic insulation. The energy absorption of aluminum foams is mostly dominated by the long stress plateau stage, leading to a low loading transmission to the protected structures. In order to more efficiently deal with more complicated requirements, functionally graded aluminum foams (FGs) have been suggested in recent decades [1]. Great efforts have been conducted to prepare FGs through metallurgy, casting, and melt foaming processes [2,3,4,5,6]. The gradient can be acquired by adjusting the cell size or fabricating different material layers [7,8,9]. FG aluminum foams have pronounced better impact dissipation performance than those with a uniform density [10,11]. Among all methods, direct foaming is more feasible and less costly to get considerable dimensions of density graded aluminum foam. As a consequence, the mechanical behavior of FGs have been experimentally and theoretically investigated [12,13,14]. The typical response of FGs was characterized the gradual or stepwise increased plateau stress which can help to tailor special structural response. 

However, this research largely focused on the FGs’ longitudinal compression in the density gradient direction. Investigations on the transverse compression of FGs is scarce. Clearly, it is crucial to examine the extensive loading conditions for these materials, because the impact could be laterally carried out on the structure. In past decades, the anisotropic compressive behavior of both closed and open cell aluminum foams has been widely specified [15,16,17,18,19,20]. Results show that the collapse strength of aluminum foam under transverse compression reduced as compared to longitudinal compression, and that the reduction was directly in relation to the cell aspect ratio. However, the reported anisotropy was substantially attributed to the elliptical cell shape along the foaming direction. This anisotropy could in fact be avoided by controlling both the preparation technology and the preferred selection in productions with uniform cell structures. 

As for the transverse compression of FGs, the mechanical response is inevitably influenced by the varied density in the lateral direction, exhibiting different deformation mechanisms. While the longitudinal compression of FGs have been studied, the mechanical behaviors under transverse compression are yet to be adequately identified. To the best of our knowledge, available studies do not provide abundant information for this problem. In this paper, FGs were prepared via a controlled foaming process by dispersing different foaming agent contents in different depths of matrixes. The anisotropy of mechanical properties and deformation mechanisms were also identified, which should help provide suggestions in structural design.

## 2. Materials and Methods 

**Figure 1:**
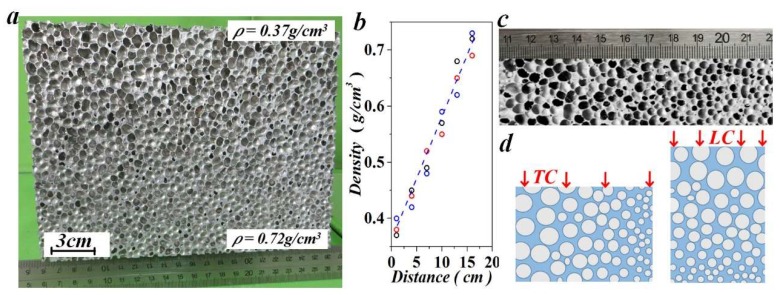
Variation trend of (**a**–**c**) cell structure and density of functionally density graded (FG) aluminum foam and (**d**), the illustration for the transverse compression (TC) and longitudinal compression (LC).

Density graded aluminum foam was produced by controlling the contents of a foaming agent, and a subsequent cooling procedure, as suggested in [8]. Firstly, pure aluminum was melted at ~680 °C. Secondly, 2 wt.% calcium was introduced as thickening agent. Thirdly, the 370 °C pre-heated TiH_2_ powders with different contents (1.0, 1.5 and 2.0 wt.%) were dispersed from bottom to the top of the molten body, acting as foaming agent. At last, a cooling procedure was implemented by spraying water at the bottom of the mold before the melt grown up. The growth process of cells was simultaneously influenced by the gravity of the melt and the rapidly reducing temperature, resulting in gradually varied cell sizes in the vertical direction. All uniaxial compression specimens were cut from blocks (200 × 200 × 160 mm^3^) where the cellular structure is quite homogeneous at specific density levels (see Figure 1a). To acquire the density gradient, series of cuboid sections in different depths from the top were cut out, and the densities were calculated. The relative density was expressed as *ρ_r_ = ρ_f_/ρ_m_*, where *ρ_f_* and *ρ_m_* are the densities of the foam and the aluminum matrix, respectively. The density nearly increased linearly with depth, as shown in Figure 1b. The variation of the cell structure with the depth is illustrated in Figure 1c. 

**Figure 2:**
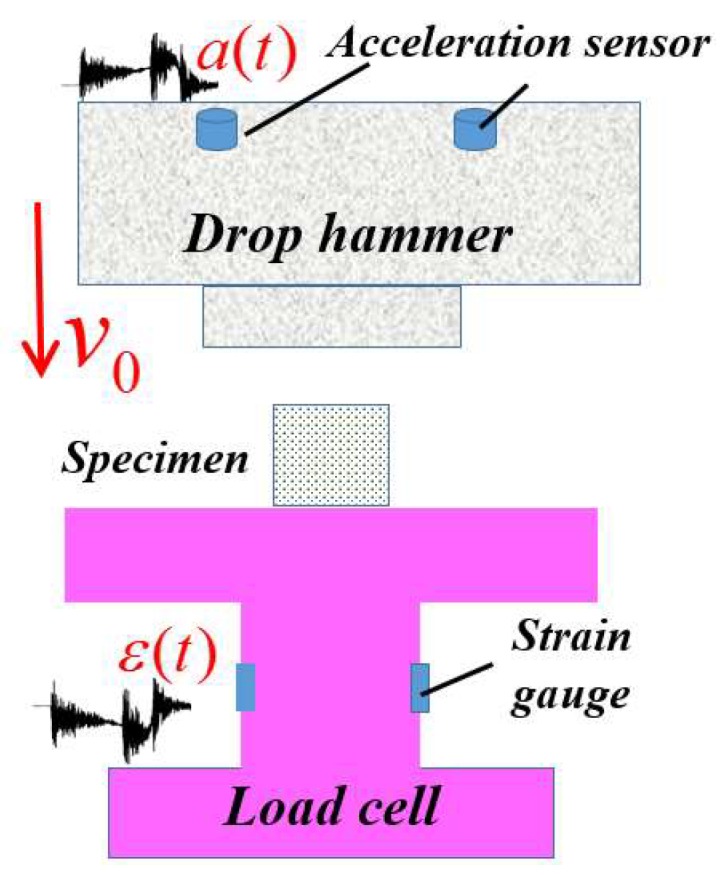
Sketch map of drop-weight tests.

Specimens with a uniform density (UF) had a nominal dimension of 40 × 40 × 40 mm^3^. Five density levels (0.39–0.65g/cm^3^) were tested to acquire the mechanical properties of UFs. The results reported here were averaged from three repeated tests for each case. For FGs, three sets of samples with a nominal density of 0.45, 0.57 and 0.62 g/cm^3^ were tested, all with a dimension of 50 × 60 × 80 mm^3^. The longitudinal and transverse compressions were respectively marked as LC and TC, as illustrated in Figure 1d. Low-velocity impact tests were carried out at room temperature by using a drop-weight impact testing system, as illustrated in Figure 2. 

The force–time and displacement–time histories in drop-weight tests were acquired by recording the acceleration of the drop hammer. The engineering strain and stress of specimens were obtained through the following calculation method:

(1) $$\varepsilon (t)=\frac{1}{L}\int_{0}^{\tau }[v_{0}-\int_{0}^{\tau }a(t)dt]dt$$

(2) $$\sigma (t)=\frac{Ma(t)}{A}$$

where *L* and *A* is height and sectional area of the specimens, respectively. *M* is the weight of the drop hammer, *a(t)* is the acceleration signal, *v_0_* is the impact velocity of the drop-weight upon the specimens, which was determined by the freefall height of the weight. A large drop hammer of 70 kg was used to reduce high frequency oscillation of the acceleration signal. The impact velocity of drop-weight under freefall from a height of 1.8–2.0 m onto the specimens was 5.94–6.26 m/s. A load cell comprising a set of semiconductor strain gauges was used to measure the impact force transmitted at the back surface of specimen (see Figure 2). In addition, the deformation processes of specimens were captured by a high-speed camera with a frame rate of 5000 fps.

**Figure 3:**
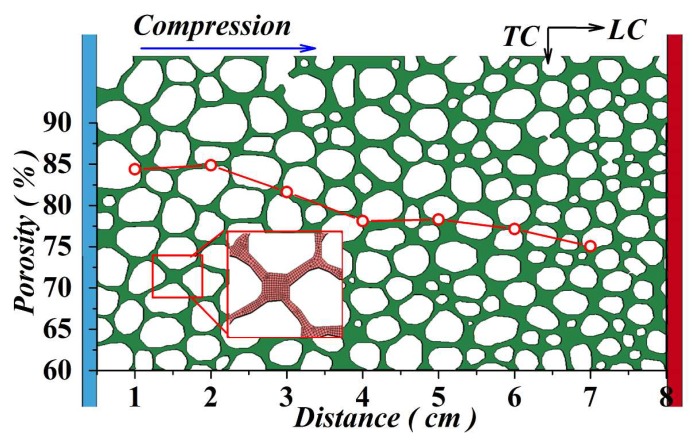
Numerical simulation model of the FG specimen and the variation trend of material porosity.

In order to acquire an insight view on the deformation and densification mechanisms of FGs, a two-dimensional numerical model was established by using a surface scanning method. At first, the surface (80 × 48 mm^2^) of the FG cut by electric discharge machine (*EDM)* was well polished. Then the surface morphology was scanned. Contour lines of each cell were extracted to be an *IGES* model. Finally, the *IGES* model was imported into commercial software *ANSYS/LS-DYNA* to create the finite element model. The model was loaded by a rigid plate moving with a velocity–time history extracted from the experiments. The support at the other side was modelled by a stationary rigid plate (see Figure 3). The red line shows the variation trend of the material porosity. The red squares specify the finite mesh elements of a single connection point between four cells. 

## 3. Results

### 3.1. Quasi-Static Compression Behavior

**Figure 4:**
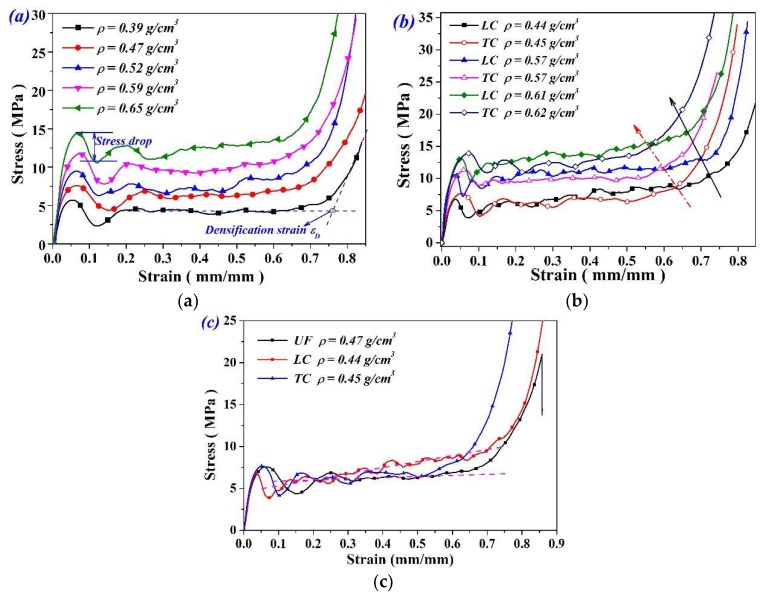
Mechanical response of (**a**) UFs and (**b**) FGs under quasi-static compression and (**c**) comparison of stress-strain response between UF and FG specimens.

Figure 4a shows a set of stress-strain curves of UFs with different densities. All curves exhibited three compression stages, including a flat stress plateau stage which primarily accounts for energy absorption. A distinct stress drop was observed after the yield stage which should be attributed to the onsets of the cell’s collapse. Compressive strength and plateau stress both increase with the density. The results of LC and TC specimens are shown in Figure 4b. Typical stress–strain responses of UF and FG specimens are compared in Figure 4c. As we can see, LC exhibits progressively increased plateau stress and lower stress drop than those of UFs. The increase trend of stress at the plateau stage are demonstrated by the dashed lines. As compared with TC specimens, three obvious characteristics of LC specimens can be found: lower compressive strength, higher plateau stress, and larger densification strain. 

### 3.2. Drop-Weight Impact Tests

**Figure 5:**
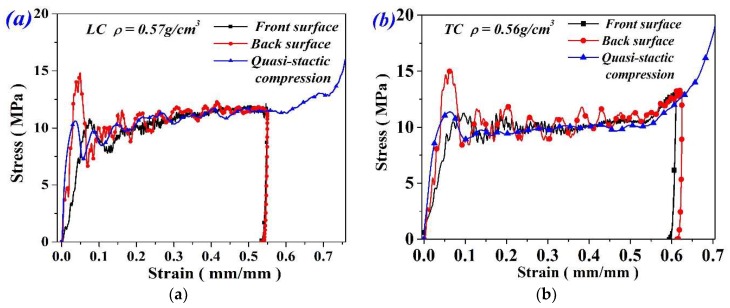
Validation of stress equilibrium for specimens under (**a**) LC and (**b**) TC.

Figure 5a,b shows typical drop-weight test results performed on FGs with nominal density of ~0.56 g/cm^3^ under longitudinal and transverse compression, respectively. The stress at the impact surface and the back surface of the specimen were both compared with the stress under quasi-static compression. As observed, at the elastic stage, the elasticity modulus under impact tests was smaller because of the data smoothing process. Also, it can be seen that the first peak force at the rear surface is higher than that at the front surface, which should be attributed to the impact effect at the interface between the load cell and the rigid support. However, after the failure strain, the stress profile of the back surface agrees well with that of the front surface. In this case, the macro deformation of the specimen could be assumed to be homogenous. As a result, the stress profiles of the front surface were adopted to characterize the dynamic compression behavior of FGs in the following contexts. 

**Figure 6:**
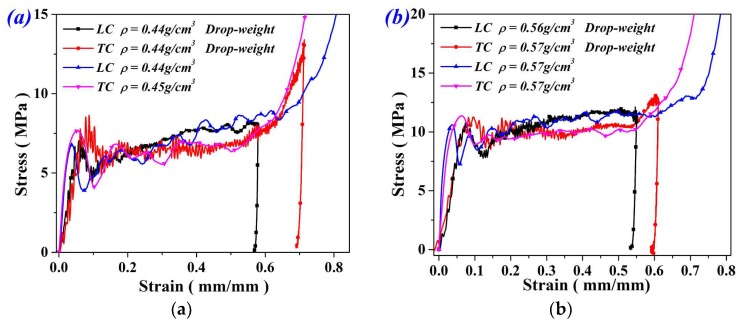
Mechanical response of (**a**) UFs and (**b**) FGs under impact compression.

Typical dynamic test results of FGs with different densities under LC and TC were indicated in Figure 6. As we can see, FGs under LC and TC show similar compressive behavior with that under static compression. FGs under TC exhibit higher strength and lower plateau stress. Also, dynamic strength and plateau stress of both LC and TC specimens agree well with the quasi-static compression values, indicating that the strain rate have no obvious effect on the mechanical properties of FGs in the present impact velocity range. For LC and TC specimens subjected to the same impact energy, TC specimens produce larger compression strain (see Figure 6). One can conclude that FGs under a longitudinal impact show better energy when absorbing performance in the present velocity range. 

### 3.3. Mechanical Properties and Energy Absorption

**Figure 7:**
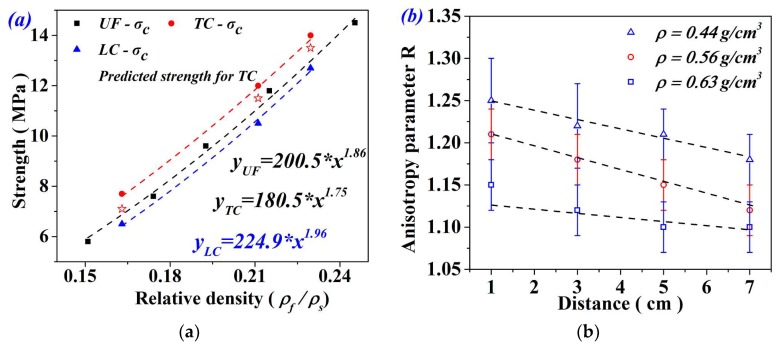
(**a**) Compressive strength of UF and FG aluminum foams and (**b**) anisotropy parameter.

The compressive strength *σ_c_* of aluminum foam under both longitudinal and transverse compression can be directly determined by the relative density [21,22]:

(3) $$\sigma_{c}=A\rho_{r}{}^{\alpha }$$

where *A* and *α* are fitting parameters. Therefore, the variation of average strengths with the density of all specimens (UF, LC and TC) was scattered in Figure 7a. The strength can be well fitted by the relation. Clearly, the strength of TC specimens is higher than that of UF and LC specimens. However, the strength of the LC specimen was slightly lower than that of UF. This can be attributed to the fact that the damage resistance of the LC specimen was dominated by the weakest part of material with the lowest density.

In the case of TC, materials with different densities experienced the same deformation rate. The strength can be calculated by averaging the load sharing of each section in the specimen. The transverse compression strength of aluminum foams can be predicted by taking cell shape anisotropy into consideration [17,19]. For three groups of FGs, the cell shape anisotropy parameter R at different depths of the specimen was measured and illustrated in Figure 7b. As we can see, the cell shape anisotropy slightly increased with cell size. Therefore, the anisotropy parameter was set as 1.22, 1.18, and 1.13 for three sets of FGs. As a consequence, the nominal strength of TC specimens was predicted as:

(4) $$\sigma_{TC}=\frac{1+1/R}{2R}*\frac{A}{L}\int_{0}^{L}{\left[\rho_{r1}+\frac{x}{L}(\rho_{r2}-\rho_{r1})\right]}^{\alpha }dx$$

where *ρ_r1_* and *ρ_r2_* are the relative density at two ends of the specimen, *L* is the sample length in density varied direction, *R* represents the cell shape anisotropy, and *A* and *α* are fitting parameters for UFs as specified in Figure 7a. The predicted values are indicated in Figure 7a, which are slightly lower than the experiment results. This is probably due to the change of deformation mechanism: transverse compression may not only change the load sharing mechanism, but also form the laterally constrained structure [23].

**Figure 8:**
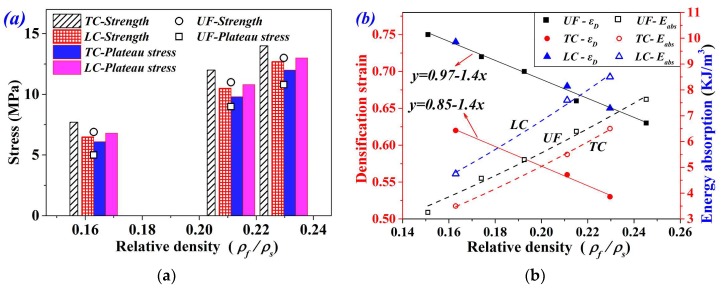
(**a**) Mechanical properties and (**b**) energy absorption of UF and FG foams.

Figure 8a shows a comparison of *σ_c_* and *σ_pl_* between LC and TC specimens, where strength and plateau stress of UFs were also scattered. It can be seen that all FGs exhibited higher plateau stress than UF foams. LC specimens exhibited the highest plateau stress and lowest strength, as compared with UF and TC specimens. For aluminum foam, energy absorption was influenced by both the plateau stress *σ_pl_* and the densification strain *ε_D_*. Therefore, the variation of *ε_D_* and energy absorption *E_abs_* were also examined (see Figure 8b). As we can see, the densification strain of LC specimens is approximate to that of UFs. However, LC exhibited better energy absorption because of their higher plateau stress. Although TC presented higher plateau stress, the energy absorption was still inferior to UFs because of the distinctly reduced densification strain. That is, transverse compression counteracted the benefit of graded density in energy absorption, though it improved the collapse resistance of materials. This should be taken into account in light-weight structural design involving FGs.

## 4. Discussion

### 4.1. Quasi-Static Deformation Mechanisms

**Figure 9:**
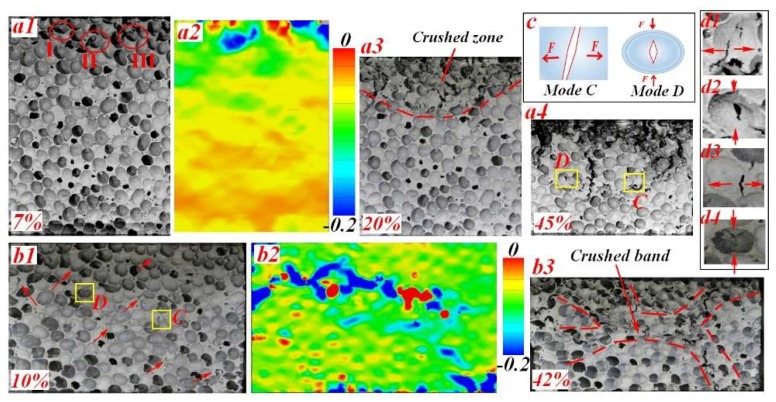
Typical deformation behavior of FG foam under (**a**) LC and (**b**) TC, (**c**) sketch of Mode C and Mode D and (**d**) typical deformation cells.

Deformation images at different stages of LC and TC specimens are shown in Figure 9. When the LC specimen was compressed over the failure strain, cell collapse first arose at the top side because of the lowest density. The corresponding compression strain map was acquired by using digital image correlation analysis [24], see Figure 9a2. With further compression, the crushed zone progressively extended downstream to the part with denser material. A gradual collapse behavior of the material is advantageous to special structural design. In the case of TC, deformation bands randomly formed in the whole region, see Figure 9b1,b2. As a consequence, the failure mode of the TC specimen was dominated by the overall shear bands, which is in accordance with UFs [25,26,27]. 

It was reported that energy absorption of aluminum foams is in relation to failure mechanism on cell/membrane scales [28,29,30,31,32]. At the cell level, three deformation mechanisms were concluded, that is, (I) large compressive deformation without rotation, (II) large deformation incorporating with obvious rotation, (III) distortion involving in-plane shear failure of cell walls [25,33]. The three mechanisms can also be specified in FGs as indicated in Figure 9a1. However, the crushed cells in LC specimens tended to localize around the moving band, while in TC specimens, the initially collapsed cells randomly distributed in the whole specimen as the red arrows indicated in Figure 9b1. 

At the membrane level, four failure patterns can be identified: bending, shearing, tearing, and a combined lateral stretching and buckling of the cell membranes, named as *Mode A–D* [34]. These failure patterns were also observed in FGs. Bending and shearing of membranes were observed at the initial collapse stage in both LC and TC. Conversely, tearing and lateral stretching, illustrated in Figure 9c, mostly arose at the later period of deformation for LC specimens. It means that *Mode C* and *D* mainly took place at higher nominal strain level [33] (see Figure 9a4). The typical cells were indicated in Figure 9d1,d2. However, in transverse compression, tearing and lateral stretching can be apparently identified even at the initial collapse stage (see Figure 9b1). The typical cells were specially indicated in Figure 9d3,d4. This can be primarily attributed to the density variation at the orientation perpendicular to the loading direction. Difference in mechanical performance of neighbouring cells lead to mismatches in deformation behavior, resulting in a larger extent of lateral strain concentration. In this case, higher strain level derived in some local regions, thus causing stretching and tearing of cell membranes. In fact, TC specimens were more likely to produce longitudinal cracking on the whole specimen, as illustrated in Figure 9b3.

### 4.2. Axial Deformation Under Drop-Weight Impact

**Figure 10:**
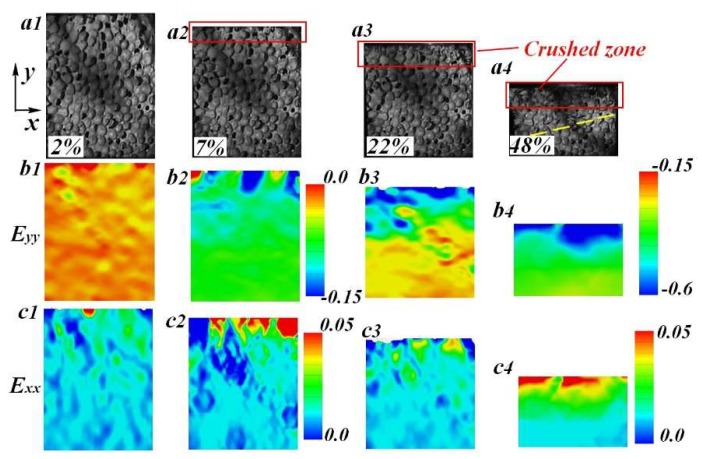
(**a**) Typical deformation behavior and (**b**–**c**) strain fields of LC specimen under drop-weight tests.

High speed images were captured during impact tests to identify the anisotropic compression behavior of FGs. A representative set of images corresponding to an LC specimen is illustrated in Figure 10. The local density increased from the top to the bottom. Axial and lateral strain maps were obtained using the digital image correlation (DIC) technique. As shown, the collapse of cells and localized deformation bands was observed at the top side of the specimen (see Figure 10b2). Afterwards, the deformation band progressively spread downstream to the region with higher density. It can be concluded that a failure pattern of LC specimen under low-velocity impact was also characterized by the gradual collapse of cells. However, when the specimen was compressed to some extent, a new deformation band formed before the front end of the crushed zone (see Figure 10a4). This should be attributed to the fact that cells in the crushed zone may not be fully compressed during the front end moving forward. Cells in this zone were partially deformed, obtaining s comparable loading capacity with uncrushed ones in the downstream. Therefore, the weakest part in the current deformed specimen may be involved in the deformation at the region outside of the crushed zone.

**Figure 11:**
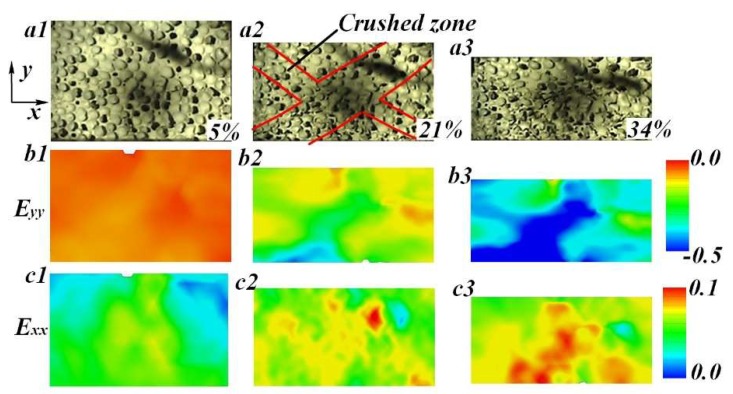
(**a**) Typical deformation behavior and (**b**–**c**) strain fields of TC specimen under drop-weight tests.

For TC specimens, multiple deformation bands randomly arose in the whole region of the specimen after the initial collapse stage (see Figure 11). Just like UFs, the weakest cells randomly were distributed into the specimen. When the specimen was compressed to the failure strain, the collapse of these cells first occurred, bringing adjacent cells into deformation and then randomly forming multiple localized regions (see Figure 11b2).

### 4.3. Lateral Deformation Under Drop-Weight Impact

**Figure 12:**
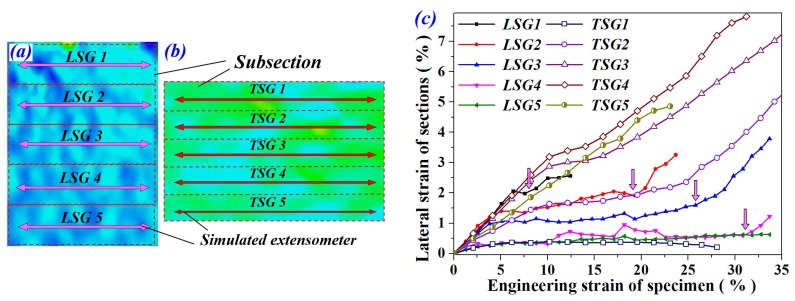
(**a**,**b**) Schematic diagram of simulated extensometers and (**c**) lateral strain curves of LC and TC specimens.

For all LC and TC specimens, intense lateral strain simultaneously arose in the fields containing crushed cells, as shown in Figure 10c1 –c4 and Figure 11c1–c3. Moreover, at the same compression extent, the specimen under TC exhibited higher lateral strain than the LC specimen. As a result, the TC specimen was more likely to produce lateral stretching failure on both macro and cell scales. In order to investigate the lateral deformation of FGs, the strain map was divided into five subsections (see Figure 12a). Five simulated extensometers were created at the mid-position of each subsection. After that, the lateral deformation of each subsection was characterized by the horizontal strain of the corresponding simulated gauge. The variation trend of lateral strain with the global compression strain of LC and TC specimens are shown in Figure 12b. It is worth mentioning that the simulated gauge failed once aluminum foam cells covered by the grids were severely crushed. Additionally, when the localized deformation band spread to the subsection, the lateral strain curve would rapidly rise up to some extent and then break off.

As shown in Figure 12c, for the LC specimen, lateral strain at different positions of the specimen exhibited regular distribution and variation trend. All curves show a plateau-like deformation stage, which corresponds to the strain state before the deformation band arrives. The red arrows in Figure 12c indicate the arrival time of deformation band. Clearly, the deformation band gradually spread from the top to the bottom, which is in accordance with the sequential images in Figure 10. 

By contrast, the distribution of lateral deformation in the TC specimen was irregular. We can see that strain of *TSG1* maintained a very low level (~0.4%) during the compression. The material in the top subsection retained intact cell morphology quite well, indicating that cells in this region mostly produced axial compression, as illustrated in Figure 11a3. The failure pattern of these cell membranes was dominated by *Mode A* and *B*. Conversely, *TSG3*~*TSG5* passed through the onset region of localized deformation band, demonstrating higher lateral strain than the other two gauges. Crushed cells concentrated into these subsections, resulting in more failure patterns of cell membranes.

Moreover, *TSG* obviously demonstrated higher lateral strain than *LSG* at the same relative position of the specimen (see Figure 12). It revealed that FGs under transverse compression produced more lateral stretching deformation on cell/membrane scales.

### 4.4. Anisotropic Compression Behavior in Numerical Simulation

**Figure 13:**
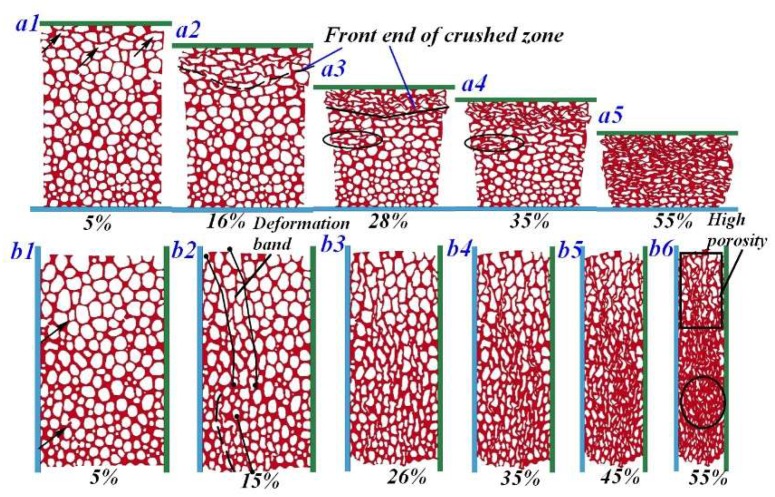
Numerical deformation behavior of FGs under (**a**) LC and (**b**) TC.

Figure 13 shows deformation images of FGs under longitudinal and transverse compression from the numerical simulation. As we can see, this compression behavior agrees well with the experiments; the deformation band gradually extended to the downstream in LC specimen and randomly occured in TC specimen. Strikingly, the deformation band more likely arose from the corner because of the stress distribution state in the rectangular specimen, as indicated in Figure 13b2. However, when the LC specimen was compressed by 28%, the other localized deformation band formed in a position far away from the front end of the crushed zone (see Figure 13a3). It revealed that localized defects and mechanical inhomogeneity of cells in the LC specimen was still the primary factor in determining the formation and evolution pattern of the deformation band. The onset of cell collapse at the initial failure stage dominated the nominal strength of the material. In the LC specimen, the initial collapse cells were all localized in the low-density region, leading to a weak resistance to failure of the material (see Figure 13a1). Conversely, in the TC specimen, the initial collapse cells were widely distributed in the specimen, therefore exhibiting a higher strength than the larger-sized cells in the LC specimen (see Figure 13b1).

In addition, when the TC specimen was compressed by 55%, materials in the high-density zone have tended to densification, while materials in the low-density zone still maintained high porosity (see Figure 13b6). The compacted zone, marked by the black circle, provided a quickly increased carrying capacity and thus lead to a densification behavior on stress-strain curve. As a consequence, the densification strain of the TC specimens was distinctly decreased. In other words, materials under transverse compression were not adequately used to absorb enough energy until the densification occurred. In summary, a lower utilization of cells and plateau stress in FG specimens under transverse compression lead to poorer energy absorption, making it more efficient than LC specimen.

## 5. Conclusions

Functionally density graded aluminum foam (FG) was prepared through a controlled foaming process by dispersing different content of foaming agent at different depths. An anisotropic mechanical behavior was identified under both longitudinal and transverse compression. The conclusions are summarized as follows:

1. The transverse compression of FGs lead to higher collapse strength but significantly reduced energy absorption, which was attributed to the decrease in plateau stress and densification strain.
2. The deformation behavior of FGs under longitudinal and transverse compression was respectively dominated by the progressively extended and randomly distributed deformation bands.
3. The high strength of transverse compressed specimen was primarily due to the density anisotropy and transition in deformation mechanisms at both macro and cell levels.
4. The decrease in densification strain of FGs under transverse compression was caused by lower utilization of cells in the high porosity zone.
5. Transverse compression caused more tearing and lateral stretching deformation on cells because of the high lateral strain level in the specimens.
